# Polymeric Materials for Wastewater Treatment Applications

**DOI:** 10.3390/polym17040552

**Published:** 2025-02-19

**Authors:** Marta Otero, Ricardo N. Coimbra

**Affiliations:** 1 Departamento de Química y Física Aplicadas, Universidad de León, Campus de Vegazana s/n, 24071 León, Spain; 2 Department of Environment and Planning, University of Aveiro, Santiago Campus, 3810-193 Aveiro, Portugal

## 1. Introduction

Water of adequate quality is crucial for the survival of most life forms, playing a key role in human health, social and economic progress, and the functioning of ecosystems [1,2,3,4]. Still, only 2.5% of the Earth’s water is freshwater, and less than 1% of that is easily accessible [5,6]. This availability is further threatened by climate change and human activity **[7,8,9]**. Additionally, the growth of populations and industries continues to drive the ongoing deterioration of freshwater quality worldwide, putting its sustainability at risk [10,11,12]. Given these challenges, ensuring safe and sufficient water supplies for all is one of the Sustainable Development Goals (SDGs) set by the United Nations General Assembly in 2015 with a target for 2030 [13,14,15]. Achieving this goal requires the development and implementation of effective wastewater treatment solutions [16,17,18].

Urban wastewater is a major contributor to water pollution if not properly collected and treated [19]. It is often contaminated with bacteria, viruses, harmful chemicals—including micropollutants—and excessive nutrients, which, if left untreated and released into the environment, pose risks to human health and harm rivers, lakes, and coastal waters [20,21,22,23]. Taking the example of the European Union (EU), the adoption of the Urban Wastewater Treatment Directive in 1991 (Directive 91/271/EEC) allowed for the improvement of the quality of European rivers, lakes, and seas [24,25,26,27]. Despite its success, more than 30 years later, an update built on those achievements was necessary [28,29,30]. An updated Urban Wastewater Treatment Directive (UWWTD) [31], which entered into force on 1 January 2025, addresses ongoing pollution issues, as well as new challenges in managing urban wastewater [32]. This UWWTD [31] aligns with European environmental strategies, including the European Green Deal [33], the Zero Pollution Action Plan [34], and the Circular Economy Plan [35]. Among the new regulations, the UWWTD [31] mandates the following: (i) collection and treatment of wastewater in all urban areas of more than 1000 inhabitants; (ii) more exigent limits on the removal of nutrients with appropriate tertiary treatment; (iii) removal of micropollutants with quaternary treatment, financed through extended producer responsibility by the sectors responsible for the pollution; (iv) ensuring that treatment plants are energy-neutral and reducing their greenhouse gas emissions by 2045. Similar regulations aiming to protect the environment and human health have already been or will soon be in force across different world regions.

Compliance with increasingly demanding discharge limits makes necessary the implementation of advanced water treatments and the development of efficient processes and materials [36,37,38]. In this sense, polymers offer remarkable properties and capacities so their applications in water treatment are a growing research topic [39,40,41,42]. Researchers from various fields have explored and/or developed traditional and innovative processes, highlighting the significant potential of polymeric materials in removing undesired substances, organisms, and pollutants of diverse origins from wastewater, managing sludge disposal, recycling materials, and enhancing the efficiency and cost-effectiveness of water treatment processes.

In the above-referred context, the published Special Issue (SI) and the present editorial aim to highlight the relevant role that polymers and polymeric materials may play in the conservation of the aquatic environment, namely, by their application in water treatment. Nineteen manuscripts were submitted to this Special Issue and twelve were finally published. The following section is an overview of the latter, summarizing their main contributions and novelties.

## 2. Overview of Published Articles

Among the published manuscripts in the SI, there were ten research works and two review papers. The latter included a review on the removal of microbial contamination [43] and another on a support layer for solar-driven interfacial steam generation (SISG) application to water treatment [44]:

The work by Akinsemolu and Onyeaka [43] reviewed the published literature on the application of hydrogel polymers, which are networks of insoluble hydrophilic polymers (either natural or synthetic) that can hold large water volumes without dissolving, for microbial control in water treatment systems. The synthesis, structure, and properties of hydrogel polymers received special attention together with the mechanisms for the removal of pathogens from wastewater. It was evidenced that apart from antimicrobial capacity, biodegradability, living cell compatibility, sensitivity to environmental stimuli, and mechanical strength are important properties for the increased overall performance of hydrogels in wastewater treatment.

Yan et al. [44] presented a literature review on the existing progress in the field of multi-layer interface evaporators based on various polymers and biomaterials, along with their advantages and disadvantages for SISG water treatment processes, which has gained attention due to its low energy consumption, simple operation, and eco-friendliness. The typical multi-layer SISG evaporator consists of a photothermal layer and a support layer, with the latter playing a key role in thermal management, stability, and water transport to the evaporation interface. Although less studied than the photothermal layer, the significance of the support layer is highlighted in this review, which summarizes advancements in materials like polymers (foams and gels) and biomaterials (natural plants and carbonized materials) and discusses the structural design strategies for the support layer, emphasizing its role in enhancing the efficiency of the SISG. It is pointed out that unmodified polymers and biomaterials are simple to prepare but lack durability in harsh conditions, while modified support layers show improved performance, though scaling them for large-scale applications remains challenging. The review concludes with potential future research directions and applications of support layer materials to address global water challenges.

Regarding the research papers, two studied desalination water treatments, either by membrane filtration [45] or by evaporation on natural and synthetic fabrics [46]:

Abdelrazeq and Khraisheh [45] investigated the effect of polystyrene membrane porosity on desalination performance by direct contact membrane distillation (MD), which is a thermal-based process with high potential for water treatment. For such a purpose, the relationship between permeate flux and feed temperature in MD using polystyrene membranes with varying porosities (77%, 89%, and 94%) was assessed under pilot-scale operation. Increasing membrane porosity by 15% was found to result in a 14.6% rise in thermal efficiency and a 5% increase in evaporation efficiency. Indeed, efficiency in the rejection of dissolved solutes was significantly reduced for porosities below 89%. The work included mathematical validation and computational predictions, providing insights into the impact of membrane porosity on thermal and evaporation efficiencies in DCMD.

López-Borrell et al. [46] examined evaporation behavior from saturated NaCl and CuSO_4_·5H_2_O solutions using natural fabrics (jute (Jut), bamboo (Bam), linen composed of 50% polylactic acid (LPLA), and a non-woven fabric composed of 70% palm prunings, 20% lyocell, and 10% PLA (WL-T)) and synthetic fabrics (non-woven polyester (PES) and a fabric with an aramid taffeta structure (Ara)) in view of brackish water treatment under zero liquid discharge (ZLD) conditions. For NaCl, the evaporation rate increased with the number of cycles, as salt deposits on the fabrics enhanced evaporation, with polymeric fabrics, namely PES and LPLA, being the most promising for long-term use. In contrast, CuSO_4_·5H_2_O reduced fabric absorption capacity over cycles due to salt precipitation, which blocked the fabric structure, decreased interaction with the solution, and ultimately slowed evaporation. These findings serve as support for the selection of fabrics for ZLD wastewater treatment, offering potential benefits for industrial-scale applications.

Advanced oxidation processes (AOPs) for wastewater treatment and, specifically, the development, characterization, and application of novel oxidants or catalysts, were the focus of three of the research papers published in the SI [47,48,49]:

In the study by Li et al. [47], polyethylene glycol (PEG)-coated calcium peroxide nanoparticles (nCPs) were synthesized and used as oxidants to generate hydroxyl free radicals (•OH) in the presence of Fe^2+^ for the removal of glyphosate from water in a Fenton-based advanced oxidation process. A co-precipitation method using calcium chloride (CaCl_2_) as a precursor and polyethylene glycol 200 (PEG 200) as a surface stabilizer was used to obtain nCPs (40.88 nm) with high surface area (28.09 m^2^/g), which were characterized using various techniques like Fourier-transform infrared spectroscopy (FTIR), X-ray diffraction (XRD), Brunauer–Emmett–Teller (BET) surface area, dynamic light scattering (DLS), and field emission (FE) scanning electron microscopy (SEM). Under optimized conditions (pH 3.0, 0.2 g nCPs, Ca^2+^/Fe^2+^ molar ratio of 6, initial glyphosate concentration of 50 mg/L), 99.6% glyphosate and 75.1% chemical oxygen demand removal were achieved within just 75 min. The degradation of glyphosate was fast and followed the Behnajady–Modirshahla–Ghanbery (BMG) kinetic model. The results demonstrated that nCPs are a much more effective oxidant than commercial calcium peroxide (CaO_2_ (CP)) for removing glyphosate from water and promising for application in advanced wastewater treatment.

Kraft washing effluents (KWEs), whose major constituent is lignin biopolymer, usually contain high concentrations of sulfide ions (S(II)) since the kraft process uses sodium sulfide (Na_2_S). Luo et al. [48] synthesized N and Fe co-doped carbon dots (N,Fe-CDs) from citric acid, L-glutamic acid, and ferric chloride via a hydrothermal method to photocatalytically remove S^2–^ from KWE. The photocatalytic degradation of S^2–^ followed first-order kinetics, with an activation energy of 33.77 kJ/mol. The N,Fe-CDs (fluorescent nanoparticles with an average 3.8 nm diameter) were stable at temperatures up to 80 °C and could be reused for at least four cycles, retaining over 90% of their catalytic activity. In the treatment of KWE, the N,Fe-CDs reduced S^2–^ concentration from 1.19 to 0.41 mmol/L in 6 h, achieving near-complete remediation in 24 h. The photocatalyst also removed half of the chemical oxygen demand and was found to be safe at concentrations up to 200 mg/L, supporting its potential for cleaner kraft pulping processes.

Natural wood is primarily made up of cellulose, hemicellulose, and lignin, which together constitute over 90% of its total weight. Five types of natural wood of two types, namely hardwood (bass, beech, and balsa) and softwood (pine and fir) were impregnated by Wang et al. [49] with palladium nanoparticles to obtain different palladium nanoparticle-loaded natural wood membranes (PNNW membranes). These PNNW membranes were tested for their catalytic performance in the removal of dyes (methylene blue (MB), methyl orange (MO), and 4-nitrophenol (4-N)) from their single and mixed aqueous solutions. The softwoods, which have small-diameter channels and therefore water flux through them is relatively slow, exhibited higher degradation efficiency for single 4-NP compared to hardwood due to the longer contact time between the dye and catalyst. Both the hardwood and softwood showed high degradation efficiency for single MB and MO, but mixed pollutants interfered with each other, reducing catalytic performance. All the wood membranes demonstrated good degradation properties for different concentrations of MB, though high concentrations of MO and 4-NP resulted in lower efficiency.

Finally, adsorption was the most approached wastewater treatment in this SI, with five research papers [50,51,52,53,54] on the synthesis, characterization, and utilization of novel adsorbent materials for the removal of pollutants from wastewater:

EL-Ghoul and Alsamani [50] presented the design of a new adsorbent material made from a cellulosic non-woven textile grafted with two extracted biopolymers using a layer-by-layer grafting technique. The study detailed the extraction of *Suaeda fruticosa* polysaccharide (SFP), which was confirmed to have a pectin-like structure, followed by grafting SFP with carrageenan crosslinked using 1,2,3,4-butanetetracarboxylic acid. Characterization through FTIR and SEM revealed successful chemical grafting, the total filling of material micro-spaces with layers of grafted biopolymers, and a rough surface morphology of the synthesized adsorbent. The cationic dye methylene blue (MB) was selected as the target pollutant to test the synthesized adsorbent, which provided an adsorptive removal as high as 803 mg/g in batch experiments under agitation. The adsorption of MB followed pseudo-first-order kinetics and Langmuir/Temkin adsorption isotherms, indicating that the developed biosorbent has great potential for low-cost, efficient wastewater treatment.

El Kaim Billah et al. [51] produced a novel polymer bio-composite, CS/n-Hap, to remove toxic cadmium ions (Cd^2+^) from water, combining chitosan (CS) and nano-hydroxyapatite (n-Hap). The structure and composition of CS/n-Hap were confirmed through XRD and FT-IR analysis, which showed that both CS and n-Hap retained their characteristic features. Batch adsorption experiments under agitation revealed that CS/n-Hap outperformed pristine CS in removing Cd^2+^, with a maximum uptake of 127 mg/g under optimized conditions. The adsorption of Cd^2+^ onto CS/n-Hap was endothermic and spontaneous and followed pseudo-second-order kinetics and Freundlich isotherm equilibrium, suggesting the formation of chemical bonds between Cd^2+^ and CS/n-Hap and multi-layer adsorption. Electrostatic interactions combined with chelation, ion exchange, and surface complexation were underneath the Cd^2+^ adsorption. Additionally, CS/n-Hap was successfully regenerated and reused with only a 3% reduction in Cd^2+^ removal after five cycles, highlighting its potential for eco-friendly and cost-effective water treatment.

Klaus et al. [52] prepared a series of acrylamide-based hydrogels and then hydrogel composites with powdered activated carbon (PAC), characterized the obtained materials, and assessed their ability to adsorb per- and polyfluoroalkyl substances (PFASs). Physicochemical characterization using FTIR, thermogravimetric analysis (TGA), and swelling studies confirmed successful PAC incorporation in the composites and revealed how crosslinking density influenced the swelling ratio. Also, surface analysis showed carbon-rich areas in the composite. The removal of PFAS at relevant concentrations was analyzed by liquid chromatography–mass spectrometry (LC-MS/MS), with the hydrogel composite achieving up to 98% removal for PFOS and 96% for PFOA. The adsorption capacity of hydrogel composites was larger than that of hydrogels but lower than that of PAC. However, contrary to PAC, hydrogel composites showed high selectivity and tunable properties to optimize their performance, pointing to their significant potential as advanced materials for selective PFAS removal from water.

The study by Ojembarrena et al. [53] proved that the adsorption performance of cellulose nanofibers (CNF), which are already known for their high specific surface area and active groups, may be enhanced by hydrophobization. In this work, CNFs obtained by TEMPO-mediated oxidation followed by mechanical disintegration was hydrophobized with methyl trimethoxysilane (MTMS) and tested for the adsorptive removal of hexavalent chromium (Cr^6+^) from wastewater. Optimization was performed by adjusting the MTMS dosage for hydrophobization and operational parameters, namely contact time, pH, the initial concentration of Cr^6+^, and adsorbent dosage. Hydrophobized CNFs with 1.5 mmol MTMS/g at dosages above 500 mg/L achieved nearly complete Cr^6+^ removal (<97%), with the best performance at pH 3. The batch adsorption results obtained under stirring fitted pseudo-second-order kinetics at low concentrations and intraparticle diffusion at higher concentrations and Freundlich equilibrium.

Ye et al. [54] explored the conversion of polystyrene (PS) plastic waste into sorbent foam via a simple sulfonation process, producing sulfonated polystyrene (SPS) foams with a porous, hydrophilic surface and abundant sulfonic groups for effective pollutant binding. SPS was tested for the adsorption of Pb^2+^, lysozyme, and MB in batch, filtration, and cyclic experiments, with Pb^2+^ and lysozyme fitting the Langmuir model and maximum adsorption capacities of 10.5 mg/g and 15.7 mg/g, respectively, while MB adsorption followed the Freundlich isotherm. Despite lower adsorption capacities compared to PS-based sorbents in the literature, SPS’s bulky self-standing morphology allowed easy post-use separation. SPS was easily regenerated by acid washing and showed excellent cyclic performance. Due to its abundant feedstock, simple preparation, and effective regeneration, SPS foams were highlighted as promising sorbents for wastewater treatment.

## 3. Challenges and Future Perspectives

The published papers in this SI provide a thorough understanding of the application of polymeric materials in wastewater treatment. A wide range of important topics was covered, and the latest research findings on different types of treatments, from desalination membranes to hydrogels, catalysts, absorbers, and adsorbents for the removal of different types of contaminants were assessed. These assessments were derived either from the comprehensive literature reviews or from practical materials development and/or engineering applications, reflecting concern about the decontamination of water.

Apart from the relevant information, findings, technical basis, or practical guidance provided in this SI, challenges and necessities for future research were raised.

Akinsemolu and Onyeaka [43] emphasized in their review the need for further research to optimize hydrogel polymers for pathogen removal from wastewater, focusing on assessing their efficiency against specific microorganisms and comparing the performance and cost of reused versus new polymers. Overall, refining hydrogel formulations to improve selectivity, long-term stability, and scalability, thereby contributing to cleaner water and more sustainable environmental management, are key aims for future research on hydrogels [55]. For its part, the review by Yan et al. [44] highlighted the challenges in designing support layers for photothermal evaporators, stressing the need for broader material options, enhanced performance, and commercial viability to improve SISG technology for sustainable water purification. For this purpose, and in view of synergistic applications in water purification, exploring hybrid photothermal materials should be considered [56].

The findings by Abdelrazeq and Khraisheh [45] constitute a relevant basis for the optimization of MD processes for wastewater treatment, but further studies are needed to increase energy efficiency for large-scale applications. Despite the advantages of polymeric membranes, namely enhanced processability, low cost, polymer variability, and availability, challenges remain for MD’s large-scale application, including high energy consumption, flux decay related to fouling, and economic feasibility [57]. The study by López-Borrel et al. [46] proved the applicability of polymeric fabrics for brackish ZLD water treatment. In line with recent advances [58], more salts apart from the studied ones (NaCl and CuSO_4_·5H_2_O) need to be tested, operational conditions should be optimized, and scaling assessed to support practical application in view of ZLD.

Li et al. [47] developed an efficient oxidant for advanced wastewater treatment via the Fenton process, achieving nearly complete glyphosate removal with 75% mineralization. Besides the analysis of transformation products, further testing on other organic micropollutants and real matrices is needed for practical implementation. Luo et al. [48] assessed the applicability of their N,Fe-CDs for the removal of S^2−^ from real matrices (KFE) at the laboratory scale, with upscale studies needed for real applications. The authors highlighted that more research on N,Fe-CDs could help in the catalytic removal of odorous sulfur-containing organic compounds produced during kraft pulping (e.g., CH_3_SCH_3_ or CH_3_SSCH_3_). On the other hand, the results obtained by Wang et al. [49] on their PNNW membranes highlighted the need for tailored wood-based membranes depending on the pollutants in water, with the efficient catalytic degradation of mixed pollutants from real complex matrices being a main challenge. In general, AOP applications in water treatment face several challenges, such as the short lifespan of free radicals, high reagent consumption, and secondary pollution risks [57]. On the other hand, the efficiency of AOP is highly affected by operational parameters and matrix effects, which need to be assessed for an optimized performance [59].

A simple and novel low-cost polymeric biosorbent material was designed by EL-Ghoul and Alsamani [50] and tested for MB cationic dye adsorptions. An efficient bio-composite was developed by El Kaim Billah et al. [51], which was shown to be efficient for the adsorptive removal of Cd^2+^. The acrylamide-based hydrogel composites with PAC developed by Klaus et al. [52] are very efficient in the removal of environmentally relevant concentrations of PFOs and PFAS. Hydrophobized CNFs hydrogel produced by Ojembarrena et al. [53] provided better adsorption than CNFs and cellulose nanomaterials for the removal of Cr^6+^. Along with that, Ye et al. [54] obtained interesting results on the utilization of SPS, whose production is in line with the principles of circular economy, for the adsorption of Pb^2+^, lysozyme, and MB. Future challenges to expand the application of polymeric advanced adsorbent materials include the following [41,60,61]: (i) the assessment of their potential in the removal of different toxic metals, dyes, pesticides, pharmaceuticals, etc.; (ii) exploring their selectivity and competitive effects in complex real matrices; (iii) carrying out pilot and full-scale experiments to support their practical applicability; (iv) developing protocols for their synthesis and performance evaluation; (v) assessing reusability; (vi) developing multifunctional polymeric materials; and (vii) integrating adsorption processes with advanced remediation technologies. On the basis of research and innovations devoted to upgrading performance, selectivity, and sustainability, polymeric adsorbents are set to play a key role in sustainable water treatment [41].

## References

[1] Vardon M.J., Le T.H.L., Martinez-Lagunes R., Pule O.B., Schenau S., May S., Grafton R.Q. (2025). Accounting for Water: A Global Review and Indicators of Best Practice for Improved Water Governance. Ecol. Econ..

[2] Yan T., Shen S.L., Zhou A. (2022). Indices and Models of Surface Water Quality Assessment: Review and Perspectives. Environ. Pollut..

[3] Ngatia M., Kithiia S.M., Voda M., Ssembajwe R. (2024). Spatial and Temporal Variations in Surface Water Quality: A Continental Review. Geogr. Tech..

[4] Elmadani M., Kasmai Kiptulon E., Klára S., Orsolya M. (2024). Systematic Review of the Impact of Natural Resource Management on Public Health Outcomes: Focus on Water Quality. Resources.

[5] Suresh Isravel R., Karunamurthy K. (2023). A Review of Solar Thermal Desalination Using Nanotechnology. Materials Today: Proceedings.

[6] Mishra R.K., Dubey S.C. (2023). Fresh Water Availability and It’s Global Challenge. J. Mar. Sci. Res..

[7] Islam M.S., Mostafa M.G. (2024). Impacts of Climate Change on Global Freshwater Quality and Availability: A Comprehensive Review. J. Water Env. Technol..

[8] Kulionis V., Pfister S. (2022). A Planetary Boundary-Based Method to Assess Freshwater Use at the Global and Local Scales. Environ. Res. Lett..

[9] Khondoker M., Mandal S., Gurav R., Hwang S. (2023). Freshwater Shortage, Salinity Increase, and Global Food Production: A Need for Sustainable Irrigation Water Desalination—A Scoping Review. Earth.

[10] Hekmatnia M., Isanezhad A., Ardakani A.F., Ghojghar M.A., Ghaleno N.D. (2023). An Attempt to Develop a Policy Framework for the Global Sustainability of Freshwater Resources in the Virtual Water Trade. Sustain. Prod. Consum..

[11] Syeed M.M.M., Hossain M.S., Karim M.R., Uddin M.F., Hasan M., Khan R.H. (2023). Surface Water Quality Profiling Using the Water Quality Index, Pollution Index and Statistical Methods: A Critical Review. Environ. Sustain. Indic..

[12] Rahman A., Kumar P., Dominguez F. (2022). Increasing Freshwater Supply to Sustainably Address Global Water Security at Scale. Sci. Rep..

[13] Asante-Darko D., Dadzie S.A., Kwarteng A., Agbodjah S., Aryee T.E. (2024). Effects of Circular Economy Practices and Accounting Innovations on Sustainable Development Goals. Circ. Econ. Sustain..

[14] Garcia C., López-Jiménez P.A., Pérez-Sánchez M., Sanchis R. (2024). Methodology for Assessing Progress in Sustainable Development Goals Indicators in Urban Water Systems. How Far Are We from the 2030 Targets?. Sustain. Cities Soc..

[15] Ojeda-Matos G., Jones-Crank J.L. (2025). The Potential of the Water-Energy-Food Nexus Approach in Advancing the Sustainable Development Goals: A PRISMA-Based Systematic Review. Env. Sci. Policy.

[16] Jones E.R., Bierkens M.F.P., Wanders N., Sutanudjaja E.H., van Beek L.P.H., van Vliet M.T.H. (2022). Current Wastewater Treatment Targets Are Insufficient to Protect Surface Water Quality. Commun. Earth Env..

[17] Saadatinavaz F., Alomari M.A., Ali M., Saikaly P.E. (2024). Striking a Balance: Decentralized and Centralized Wastewater Treatment Systems for Advancing Sustainable Development Goal 6. Adv. Energy Sustain. Res..

[18] Sanei E., Gómez-Gallegos M.A., Márquez I., Segovia-Hernández J.G., Ramírez-Corona N., Aristizábal-Marulanda V. (2024). Chapter 4: Advances in Wastewater Treatment Technologies as Enablers to Reach Sustainable Development Goal 6. Contributions of Chemical Engineering to Sustainability.

[19] Villez K., Aguado D., Alferes J., Plana Q., Ruano M.V., Samuelsson O. (2024). Metadata Collection and Organization in Wastewater Treatment and Wastewater Resource Recovery Systems.

[20] Carneiro R.B., Nika M.C., Gil-Solsona R., Diamanti K.S., Thomaidis N.S., Corominas L., Gago-Ferrero P. (2024). A Critical Review of Wastewater-Based Epidemiology as a Tool to Evaluate the Unintentional Human Exposure to Potentially Harmful Chemicals. Anal. Bioanal. Chem..

[21] Elehinafe F.B., Agboola O., Vershima A.D., Bamigboye G.O. (2022). Insights on the Advanced Separation Processes in Water Pollution Analyses and Wastewater Treatment—A Review. S. Afr. J. Chem. Eng..

[22] Mohammadi S.A., Najafi H., Zolgharnian S., Sharifian S., Asasian-Kolur N. (2022). Biological Oxidation Methods for the Removal of Organic and Inorganic Contaminants from Wastewater: A Comprehensive Review. Sci. Total Environ..

[23] Alshami A., Ali E., Elsayed M., Eltoukhy A.E.E., Zayed T. (2024). IoT Innovations in Sustainable Water and Wastewater Management and Water Quality Monitoring: A Comprehensive Review of Advancements, Implications, and Future Directions. IEEE Access.

[24] Källqvist T., Molver J., Oug E., Berge D., Tjomsland T., Johansen S.S. (2002). Implementation of the Urban Waste Water Treatment Directive in Norway—An Evaluation of the Norwegian Approach Regarding Wastewater Treatment.

[25] Pistocchi A., Dorati C., Grizzetti B., Udias A., Vigiak O., Zanni M. (2019). Water quality in Europe: Effects of the Urban Wastewater Treatment Directive. A retrospective and scenario analysis of Dir. 91/271/EEC, EUR 30003 EN, Publications Office of the European Union, Luxembourg. https://publications.jrc.ec.europa.eu/repository/handle/JRC115607.

[26] Preisner M., Smol M., Szołdrowska D. (2021). Trends, Insights and Effects of the Urban Wastewater Treatment Directive (91/271/EEC) Implementation in the Light of the Polish Coastal Zone Eutrophication. Env. Manag..

[27] Smith A. (2000). Fitting in with Brussels: Implementing the Urban Waste Water Treatment Directive in England and Wales. J. Environ. Policy Plann..

[28] Prochaska C., Zouboulis A. (2020). A Mini-Review of Urban Wastewater Treatment in Greece: History, Development and Future Challenges. Sustainability.

[29] Badhofer A. (2023). Quaternary Treatment According to the Proposal for a Recast of the EU Urban Wastewater Treatment Directive and Its Monetary Implications for Candidate Countries (e.g., Serbia). Master’s Thesis.

[30] Commission Staff (2022). Working Document Impact Assessment Accompanying the Document “Proposal for a Directive of the European Parliament and of the Council Concerning Urban Wastewater Treatment (Recast)”.

[31] The European Parliament and the Council of the European Union (2024). Directive (EU) 2024/3019 of the European Parliament and of the Council of 27 November 2024 Concerning Urban Wastewater Treatment (Recast) (Text with EEA Relevance).

[32] Kardos M.K., Patziger M., Jolánkai Z., Clement A. (2025). The New Urban Wastewater Treatment Directive from the Perspective of the Receiving Rivers’ Quality. Env. Sci. Eur..

[33] European Commission (2019). The European Green Deal.

[34] European Commission (2021). Pathway to a Healthy Planet for All Action Plan: “Towards Zero Pollution for Air, Water and Soil”.

[35] European Commission (2020). A New Circular Economy Action Plan: For a Cleaner and More Competitive Europe.

[36] Shamshad J., Ur Rehman R. (2024). Innovative Approaches to Sustainable Wastewater Treatment: A Comprehensive Exploration of Conventional and Emerging Technologies. Environ. Sci. Adv..

[37] Gedda G., Balakrishn K., Devi R.U., Shah K.J. (2021). Introduction to Conventional Wastewater Treatment Technologies: Limitations and Recent Advances. Advances in Wastewater Treatment I. Materials Research Foundations.

[38] Bairagi S., Wazed Ali S., Ul Islam S. (2020). Conventional and Advanced Technologies for Wastewater Treatment. Environmental Nanotechnology for Water Purification.

[39] Ahmaruzzaman M., Roy P., Bonilla-Petriciolet A., Badawi M., Ganachari S.V., Shetti N.P., Aminabhavi T.M. (2023). Polymeric Hydrogels-Based Materials for Wastewater Treatment. Chemosphere.

[40] Saravanan A., Thamarai P., Kumar P.S., Rangasamy G. (2022). Recent Advances in Polymer Composite, Extraction, and Their Application for Wastewater Treatment: A Review. Chemosphere.

[41] Alkhaldi H., Alharthi S., Alharthi S., AlGhamdi H.A., AlZahrani Y.M., Mahmoud S.A., Amin L.G., Al-Shaalan N.H., Boraie W.E., Attia M.S. (2024). Sustainable Polymeric Adsorbents for Adsorption-Based Water Remediation and Pathogen Deactivation: A Review. RSC Adv..

[42] Lakkimsetty N.R., Feroz S., Karunya S., Motilal L., Saidireddy P., Suman G. (2022). Synthesis, Characterization and Application of Polymer Composite Materials in Wastewater Treatment. Materials Today: Proceedings.

[43] Akinsemolu A.A., Onyeaka H. (2024). Advances in Hydrogel Polymers for Microbial Control in Water Systems. Polymers.

[44] Yan H., Wang P., Li L., Zhao Z., Xiang Y., Guo H., Yang B., Yang X., Li K., Li Y. (2024). Development Status of Solar-Driven Interfacial Steam Generation Support Layer Based on Polymers and Biomaterials: A Review. Polymers.

[45] Abdelrazeq H., Khraisheh M. (2023). Porosity Effect of Polystyrene Membranes on Desalination Performance: A Combined Experimental and Numerical Heat and Mass Transfer Study in Direct Contact Membrane Distillation. Polymers.

[46] López-Borrell A., Lora-García J., Cardona S.C., López-Pérez M.F., Fombuena V. (2024). Vapor Pressure and Evaporation Studies of Saline Solutions on Natural and Synthetic Fabrics for Industrial Water Treatment. Polymers.

[47] Li F., Choong T.S.Y., Abdullah L.C., Siti Nurul S.N.A., Amerhaider Nuar N.N. (2023). Effective Removal of Glyphosate from Aqueous Systems Using Synthesized PEG-Coated Calcium Peroxide Nanoparticles: Kinetics Study, H_2_O_2_ Release Performance and Degradation Pathways. Polymers.

[48] Luo H., Liu H., Sun C. (2023). Removal of Sulfide Ions from Kraft Washing Effluents by Photocatalysis with N and Fe Codoped Carbon Dots. Polymers.

[49] Wang Z., Jia C., Xiang H., Zhu M. (2023). Palladium Nanoparticle-Loaded Mesostructural Natural Woods for Efficient Water Treatment. Polymers.

[50] EL-Ghoul Y., Alsamani S. (2024). Highly Efficient Biosorption of Cationic Dyes via Biopolymeric Adsorbent-Material-Based Pectin Extract Polysaccharide and Carrageenan Grafted to Cellulosic Nonwoven Textile. Polymers.

[51] El Kaim Billah R., Ayouch I., Abdellaoui Y., Kassab Z., Khan M.A., Agunaou M., Soufiane A., Otero M., Jeon B.H. (2023). A Novel Chitosan/Nano-Hydroxyapatite Composite for the Adsorptive Removal of Cd(II) from Aqueous Solution. Polymers.

[52] Klaus M.V.X., Gutierrez A.M., Hilt J.Z. (2023). Development of Poly(Acrylamide)-Based Hydrogel Composites with Powdered Activated Carbon for Controlled Sorption of PFOA and PFOS in Aqueous Systems. Polymers.

[53] Ojembarrena F.d.B., Sánchez-Salvador J.L., Mateo S., Balea A., Blanco A., Merayo N., Negro C. (2022). Modeling of Hexavalent Chromium Removal with Hydrophobically Modified Cellulose Nanofibers. Polymers.

[54] Ye C., Pan Z., Shen Y. (2022). Facile Conversion of Polystyrene Waste into an Efficient Sorbent for Water Purification. Polymers.

[55] Visan A.I., Negut I. (2025). Environmental and Wastewater Treatment Applications of Stimulus-Responsive Hydrogels. Gels.

[56] Tessema A.A., Wu C.M., Motora K.G., Lee W.H., Peng Y.T. (2024). A Review on State of Art of Photothermal Nanomaterials for Interfacial Solar Water Evaporation and Their Applications. Desalination.

[57] Kakkar R., Kaur D.P., Raj S. (2025). Membrane Distillation for Sustainable Water Desalination: A Review of Principles, Materials, and Applications. Water Air Soil. Pollut..

[58] Scelfo G., Trezzi A., Vassallo F., Cipollina A., Landi V., Xenogianni C., Tamburini A., Xevgenos D., Micale G. (2025). Demonstration of Ultra-High-Water Recovery and Brine Concentration in a Prototype Evaporation Unit: Towards Zero Liquid Discharge Desalination. Sep. Purif. Technol..

[59] Khan Q., Sayed M., Khan J.A., Rehman F., Noreen S., Sohni S., Gul I. (2024). Advanced Oxidation/Reduction Processes (AO/RPs) for Wastewater Treatment, Current Challenges, and Future Perspectives: A Review. Environ. Sci. Pollut. Res. Int..

[60] Mazumder M.A.J. (2023). Polymeric Adsorbents: Innovative Materials for Water Treatments. Curr. Anal. Chem..

[61] Saleh T.A. (2021). Protocols for Synthesis of Nanomaterials, Polymers, and Green Materials as Adsorbents for Water Treatment Technologies. Environ. Technol. Innov..

